# Fine-scale mapping in case-control samples using locus scoring and haplotype-sharing methods

**DOI:** 10.1186/1471-2156-6-S1-S74

**Published:** 2005-12-30

**Authors:** Keith Humphreys, Mark M Iles

**Affiliations:** 1 Medical Epidemiology and Biostatistics, Karolinska Institutet, PO Box 281, S-171 77 Stockholm, Sweden

## Abstract

Both haplotype-based and locus-based methods have been proposed as the most powerful methods to employ when fine mapping by association. Although haplotype-based methods utilize more information, they may lose power as a result of overparameterization, given the large number of haplotypes possible over even a few loci. Recently methods have been developed that cluster haplotypes with similar structure in the hope that this reflects shared genealogical ancestry. The aim is to reduce the number of parameters while retaining the genotype information relating to disease susceptibility. We have compared several haplotype-based methods with locus-based methods. We utilized 2 regions (D2 and D4) simulated to be in linkage disequilibrium and to be associated with disease susceptibility, combining 5 replicates at a time to produce 4 datasets that were analyzed. We found little difference in the performance of the haplotype-based methods and the locus-based methods in this dataset.

## Background

It is widely accepted that for the fine-scale mapping of disease susceptibility loci, association-based approaches are more appropriate than linkage methods. Although genome-wide association studies are often forecast, association studies currently focus predominantly on relatively small candidate regions. Such regions are suggested either by strong evidence from linkage studies or from functional arguments and are typically densely genotyped. More information about a candidate region is retained by incorporating phase in the analysis. However even in the presence of substantial linkage disequilibrium (LD) many haplotypes may exist and it has been suggested that for this reason haplotype-based studies lack power compared to 'locus-scoring' approaches [1]. Recent approaches [2,3] have sought to circumvent this problem by grouping similar haplotypes in the hope that such similarity will reflect a shared ancestry. Thus the parameter space can be reduced while, it is hoped, retaining that phase information relevant to disease susceptibility. Locus-based methods have been similarly refined to incorporate information on the estimated genealogical structure prior to formal testing [4]. The power of such approaches has been examined by Clayton et al. [5].

Using the Genetic Analysis Workshop 14 (GAW14) data we have compared the performances of several 'locus-scoring' and 'haplotype-sharing' approaches for detecting and localizing gene-trait association using case-control studies. Such methods should be extendable to nuclear family data but as yet appropriate methodology has not been developed.

## Methods

The first 3 methods we investigated are what we term 'locus-scoring' methods and do not utilize haplotype information. The last three are 'haplotype-sharing' methods and analyze the data by clustering similar haplotypes to reduce the dimensionality of the data. The 6 methods which we have implemented are denoted (i)–(vi) and are described below.

(i), (ii), (iii) Logistic regression is widely employed for the modelling of association between genes and binary traits [1,6]. Here the continuous trait *Y *is regressed on the genotypes at the diallelic loci *M *under consideration, some of which may be etiological. The *m*^th ^genotype is coded *X*_*m *_according to the number of rare alleles at the *m*^th ^locus. The model is



We implicitly assume multiplicative penetrance. We consider models with *M *= 1 (i), *M *= 6 (ii) and *M *= 6 with 5 pair-wise (adjacent loci) interactions (iii).

(iv) For sliding windows of *M *single-nucleotide polymorphisms (SNPs) Durrant et al. [3] suggest grouping similar haplotypes using hierarchical clustering. This requires calculation of a distance measure. For the case of no missing data and denoting alleles as 0 or 1, the distance between haplotypes *i *and *j *is measured as



where *p*_*m *_is the (observed) frequency of allele 1 at locus *m *and , where *I*(.) represents an indicator variable and  denotes the allele at locus *m *of the haplotype. Durrant et al. [3] recommend performing the hierarchical clustering then fitting logistic regression models using haplotype cluster membership as covariates. They search for the optimal association across different numbers of clusters and SNP window sizes and apply a Bonferroni correction.

(v), (vi) We have modified approach (iv) by considering 2 alternative measures of similarity. The similarity between a pair of haplotypes is now measured without restriction to a window of markers. The distance measure used in (v) is based upon the length of the segment shared identically by state (IBS) around a putative locus in the studied region. Distance is measured simply as 1-L_1_/L_2_, where L_1 _is the number of consecutive alleles shared either side of the putative locus, and L_2 _is the total number of markers in the region being studied. The putative locus is assumed to be located between a pair of adjacent markers. Each marker interval in the region is tested in turn as the putative locus. This is approach (v). Method (vi) modifies the distance measure used in (v) by incorporating allele frequency weights in a similar manner to (2). If *k *markers *a*, .., *a+k-1 *are shared IBS then the distance is measured as



The clustering proceeds as in method (iv). Note that we do not incorporate physical distances between markers into our measures of haplotype distances. One approach for measuring haplotype distances, incorporating marker distance information is described by Molitor et al. [7].

Other, more flexible, approaches to haplotype clustering have been proposed but are computationally demanding and have not been included here for that reason. Thomas et al. [2], for example, propose assigning haplotypes to clusters probabilistically, using the Potts model and using reversible jump Markov chain Monte Carlo (MCMC) methods to update the number of clusters and the location of the variant. This is more flexible because it allows partitions other than those formed by cutting at various points on the dendogram/genealogical tree; it instead attaches higher prior weight to more likely partitions of haplotypes.

## Results

We focused on susceptibility regions D2 and D4, simulated to be in LD. To ensure sufficient power we created four datasets of 500 cases (one affected individual from each family) and 250 controls by combining 5 replicates at a time from the Danacaa population. These datasets are referred to as Study 1 to Study 4. We used 3 locus-scoring methods. Firstly, we tested a single locus at a time, referred to as method (i). (For region D2 SNPs 1–27 refer to B03T3041 to C04R0282; and for D4 SNPs 1–38 refer to B09T8321 to B09T8360). Then, using a window covering 6 markers, we applied two variants-the first, (ii), incorporated only single-locus main effects from each of the 6 markers, and the second, (iii), additionally included pair-wise interactions between adjacent loci (11 parameters).

Haplotypes were reconstructed haplotypes from the available genotype data using maximum likelihood methods. Haplotypes were then grouped by hierarchical clustering, defining similarity either by the number of loci shared in common within the window (iv), or the maximum continuous length shared in common without weighting for allele frequency (v), and (inversely) weighting for allele frequency (vi). We tested different numbers of partitions and chose the optimal partition.

**Figure 1:**
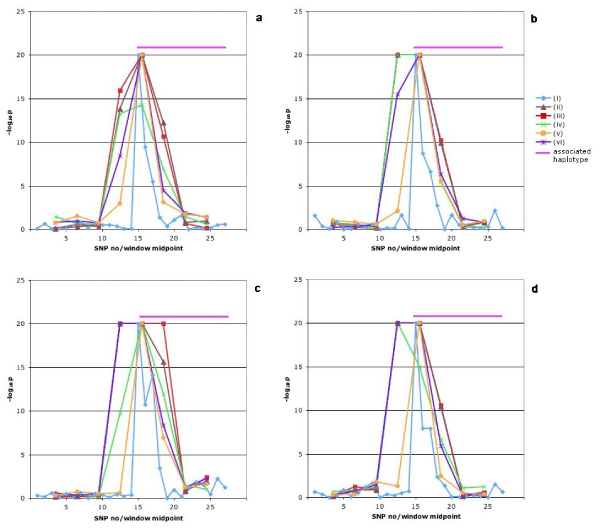
**Comparison of results for the 6 different analysis methods around the D2 region**. For the single locus method (i) the position of each locus is plotted. For the window-based methods ((ii)–(vi)) the position of the midpoint of each window is plotted. The true location of the susceptibility haplotype is indicated by the pink bar. Study 1 (a), study 2 (b), study 3 (c) and study 4 (d) are shown in the four plots.

**Figure 2:**
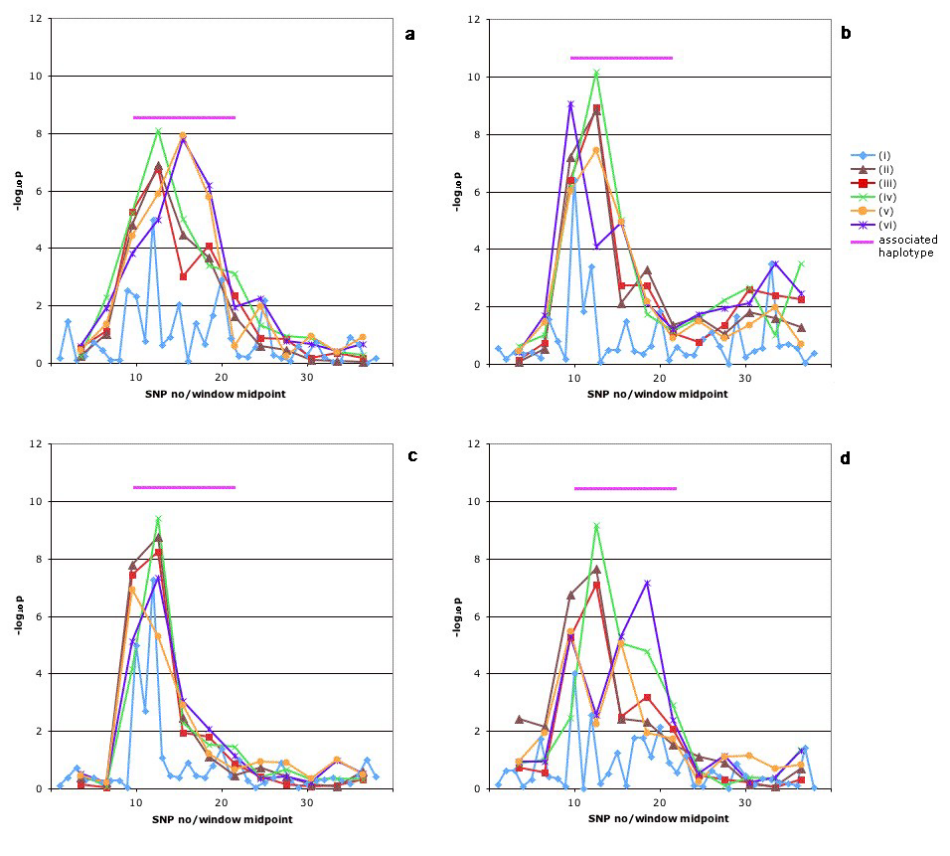
**Comparison of results for the 6 different analysis methods around the D4 region**. For the single locus method (i) the position of each locus is plotted. For the window-based methods ((ii)–(vi)) the position of the midpoint of each window is plotted. The true location of the susceptibility haplotype is indicated by the pink bar. Study 1 (a), study 2 (b), study 3 (c) and study 4 (d) are shown in the four plots.

Results for each of the different methods for LD mapping are plotted as -log_10 _*p*-values, for each of the studies, for regions D2 (Figure 1) and D4 (Figure 2). Regions harboring the disease susceptibility loci are indicated in the figures by a solid line labelled associated haplotype. Within each region we found that all groups of replicates give very similar results. In both regions there is very strong evidence for association. For each of the studies within each of the regions, all three haplotype methods ((iv), (v) and (vi)) perform similarly. Comparing haplotype-sharing with locus scoring methods, we find that haplotype-sharing methods can provide stronger evidence of association than testing a single locus at a time (i), (in particular, the D4 region). However, if the locus-scoring method uses several loci at once ((ii) and (iii)), there seems to be little difference between locus-scoring and haplotype-sharing methods, in terms of the sizes of the signals for association. We consider that it is most interesting to compare the different approaches without adjustment for multiple-testing. It is clear, even without a correction for multiple testing, that the haplotype-sharing methods do not outperform the best locus-scoring methods here. Any multiple-testing correction applied to these data would reinforce this result, but may inadvertently give the impression that the superior performance of the locus-scoring method is due to the correction. Appropriate approaches for adjusting for multiple testing that are less conservative than the Bonferroni corrections used by Durrant et al. [3], are Nyholt et al.'s procedure [8] and the permutation step-down procedure described by Westfall et al. [9]. We have also tested for gene×gene interaction effects using D2 and D4. Interactions were not detected by any of the haplotype-sharing methods (at a significance level of 0.05), but were detected in two of the four studies using the single-locus scoring method (i).

In order to better understand our results and, ultimately, the factors that determine the relative performances of the different methods, we investigated the structure of the LD in the studied regions. It is not clear how LD across the two studied regions can be formally compared. The extent of haplotype diversity can be informally judged by the proportion of theoretically possible haplotypes that are actually observed. We noticed that this proportion was markedly higher in the D4 region than it was in the D2 region. If we infer from this that the LD structure is more complex in D4 than D2, we might expect the haplotype methods to perform slightly better in D4 than in D2. We saw some evidence of this, but we recognize that the LD is confounded with the disease model, which differs between the two regions. Below we discuss possible approaches for more formally assessing the performance of LD mapping methods in relation to the LD structure of regions being studied.

## Conclusion

Although our results are not encouraging for researchers developing haplotype-sharing methods, it is important to be aware that such methods are dependent on haplotypes with similar risks having a shared ancestry, and the data considered here were not generated in such a way. Even in the D2 region, where the disease susceptibility haplotypes were chosen to be similar, a shared population history was not explicitly modelled and so it is difficult to know how well our results would generalize to real-world problems.

More thorough comparisons of the different strategies for fine mapping are needed to understand which tests are most appropriate and powerful in which situations. The types of comparisons that we have in mind are not possible using only the GAW14 simulated datasets. One approach would be to compare the performances of different methods for LD mapping when phenotypic data is simulated on the basis of different LD structures, in terms of conditional independence structures of markers in genomic regions. We are currently considering the use of log-linear models [10] for the simulation of LD structure. Log-linear models express logarithms of expected cell counts (in this case, cell counts are haplotype counts) in terms of a linear predictor including main effects and interaction terms (up to an order equal to the number of loci). We vary the highest order of interaction included in the model, as well as the strength of the interaction terms, and study the performance of the various LD mapping methods under different scenarios. The idea is not entirely new. Clayton et al. [5] have recently examined the extent to which phase is relevant to association, comparing haplotype-based and locus-based tests more formally, with the use of (linear) graphical models. They have considered some simple scenarios, such as under complete LD (where the value of Lewontin's D', but not *R*^2^, is equal to 1 between every pair of markers). The use of graphs in connection with log-linear models is described by Edwards [10]. The choice of test may ultimately be best guided by LD structure within a region, and it is hoped that the types of studies which we have described can shed some light on how to do this in practice.

## Abbreviations

GAW14: Genetic Analysis Workshop 14

IBS: Identical by state

LD: Linkage disequilibrium

MCMC: Markov chain Monte Carlo

SNP: Single-nucleotide polymorphism
